# Formations of force network and softening of amorphous elastic materials from a coarsen-grained particle model

**DOI:** 10.1038/s41598-024-59498-2

**Published:** 2024-04-17

**Authors:** Rei Kurita, Yuto Tamura, Marie Tani

**Affiliations:** 1 Department of Physics, Tokyo Metropolitan University, 1-1 Minamioosawa, Hachiouji-shi, Tokyo 192-0397 Japan; 2 Department of Physics, Kyoto University, Kitashirakawa-Oiwake-Cho, Sakyo-ku, Kyoto, 606-8502 Japan

**Keywords:** Amorphous materials, Coarsen-grain particle model, Force networks, Softening, Glasses, Nonlinear phenomena

## Abstract

Amorphous materials, such as granular substances, glasses, emulsions, foams, and cells, play significant roles in various aspects of daily life, serving as building materials, plastics, food products, and agricultural items. Understanding the mechanical response of these materials to external forces is crucial for comprehending their deformation, toughness, and stiffness. Despite the recognition of the formation of force networks within amorphous materials, the mechanisms behind their formation and their impact on macroscopic physical properties remain elusive. In this study, we employ a coarse-grained particle model to investigate the mechanical response, wherein local physical properties are integrated into the softness of the particles. Our findings reveal the emergence of a chain-like force distribution, which correlates with the planar distribution of softness and heterogeneous density variations. Additionally, we observe that the amorphous material undergoes softening due to the heterogeneous distribution of softness, a phenomenon explicable through a simple theoretical framework. Moreover, we demonstrate that the ambiguity regarding the size ratio of the blob to the force network can be adjusted by the amplitude of planar fluctuations in softness, underscoring the robustness of the coarse-grained particle model.

## Introduction

Amorphous materials, including granular substances, glasses, emulsions, foams, and cells, find widespread applications as building materials, plastics, food products, and thermal insulators in our daily lives. The mechanical properties of these materials are commonly characterized by parameters such as Young’s modulus and bulk modulus, which are pivotal for material design. Despite their adherence to classical mechanics at the level of individual compositions, these amorphous materials often display intricate macroscopic behaviors. For instance, certain components within these materials may exhibit fluid-like behavior, such as hourglass phenomena and avalanches, while also demonstrating solid-like characteristics like clogging hoppers and pipes^1–5^. Therefore, elucidating the complex mechanical responses of amorphous materials is essential for leveraging their diverse functionalities effectively.

In amorphous elastic materials, external forces propagate through localized pathways known as force networks or stress networks^6–14^. These force networks have been extensively investigated as crucial components for understanding complex dynamic responses. Over the years, force visualization experiments have been conducted to explore the mechanical behavior of granular materials^6–11^. For instance, it has been observed that compressing an unjammed state results in a shear jamming state when the force network percolates in all directions^8^. Furthermore, studies have indicated that the force network becomes isotropic through the vibration of granular materials^9^.

Despite investigations into the features of force networks, the mechanism underlying their formation remains unclear. Furthermore, the relationship between the force network and macroscopic properties, such as the bulk modulus of amorphous materials, remains ambiguous. Understanding these aspects requires studying amorphous materials on a scale larger than their individual components. For instance, the falling dynamics of grains have been likened to Rayleigh–Taylor instability phenomena in fluids^15–19^, with typical length scales of these macroscopic flows several orders of magnitude larger than the grain size. To elucidate such macroscopic behaviors, a coarse-grained model is often considered effective as a meso-scale statistical mechanics approach. This modeling technique has traditionally been employed to explain phenomenological behaviors in various systems, including polymers, liquid crystals^20^, and phase separations^21^. Recently, coarse-grained models have successfully demonstrated the fracture of amorphous materials^22,23^. In these models, it is assumed that density is heterogeneous, and the shear modulus strongly depends on density. Application of shear leads to enhanced density fluctuations, ultimately resulting in fracture.

Recent molecular dynamics simulations have revealed that the local elastic moduli exhibit spatial distribution in the random packing state^24–26^. Moreover, it has been observed that fluctuations in local elastic moduli increase as the jamming threshold is approached^25^. Additionally, features of sound wave propagation in amorphous solids have been found to correlate with the heterogeneous distribution of local elastic moduli^24^. Based on these findings, we hypothesize that the formation mechanism of force networks is also linked to the distribution of local elastic moduli. We characterize the local elastic moduli as the softness of a disk, incorporating factors such as local packing fraction, local structure, and interactions. Subsequently, we investigate the relationship between the force network and the planar distribution of softness using a coarse-grained particle model.

## Models and methods

**Figure 1:**
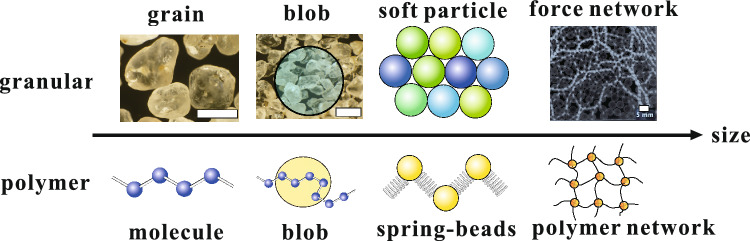
Schematic of a coarse-grained particle model for a granular material, serving as an example of amorphous elastic materials. It depicts the hierarchical structure of granular systems, which comprises individual grains, several grains forming a blob, the arrangement of these blobs, and the resulting force network. The two images on the left showcase grains, with scale bars indicating 1 mm. The image on the right visualizes the force using a photoelastic disk, with a scale bar of 5 mm. Additionally, for reference, the hierarchical structure of polymers (including blobs, spring-beads model, and polymer network in gels) is depicted, suggesting an analogy to granular systems.

We assume that microscopic physical properties should be averaged at mesoscales, such as the typical length of force networks. Accordingly, we partition the elastic material into blobs with a certain diameter. Within each blob, we characterize the local elastic moduli as softness, considering factors such as local packing fraction, local structure, and interactions refer to Fig. 1. By employing this concept, we investigated a two-dimensional soft disk model wherein the softness of each disk varies. As the blobs encompass the heterogeneous planar distribution of local packing fraction as softness, they are arranged in a close-packed hexagonal lattice configuration.

Each disk denoted as *i* possesses an elastic parameter $G_i$ indicative of its softness. A total of 65536 soft disks with a diameter *D* are organized within a triangular grid measuring 256 $\times$ 256. Periodic boundary conditions are imposed in both the *x* and *y* directions. The value of *D* is fixed at 1, while the positions of the disks along the *x* and *y* axes are indexed by the number of layers $n_x$ and $n_y$.

We generated a heterogeneous distribution of disk elasticities using the Cahn-Hilliard-Cook equation^21,27^ expressed as follows:

1 $$\begin{aligned} \frac{\partial \Gamma ({\varvec{r}})}{\partial t} = \nabla ^2[\Gamma ({\varvec{r}}) + \Gamma ({\varvec{r}})^3 - \xi ^2\nabla ^2 \Gamma ({\varvec{r}})] - \nabla \cdot \vec {g}({\varvec{r}}, t), \end{aligned}$$

where *t* denotes time, $\Gamma ({\varvec{r}})$ represents a field determining the softness of particles at position ${\varvec{r}}$ and $\xi$ stands for the correlation length of fluctuations of $\Gamma ({\varvec{r}})$. Assuming local equilibrium and employing the fluctuation-dissipation relation, the correlation function of the dimensionless noise $g({\varvec{r}}, t)$ can be described as

2 $$\begin{aligned} \langle \vec {g}_i ({\varvec{r}}, t)\vec {g}_j ({\varvec{r^{\prime }}}, t^{\prime }) \rangle = \delta _{ij}\delta ({\varvec{r}} - {\varvec{r^{\prime }}} )\delta (t - t^{\prime }), \end{aligned}$$

where $i, j = x, y$, $\delta _{ij}$ is Kronecker delta and $\delta ({\varvec{r}})$ denotes a delta function. We conducted numerical simulations of Eq. (1) on a 256 $\times$ 256 square lattice. As the order parameter is conserved, $\langle \Gamma ({\varvec{r}}) \rangle$ remains 0. Each lattice point $(n_x, n_y)$ corresponds to a blob located at that position. The relationship between $\Gamma ({\varvec{r}})$ and $G_i$ is defined as $G_i = \langle G_i \rangle + \Delta \Gamma ({\varvec{r}})/ (\Gamma _{max}-\Gamma _{min})$, where $\langle G_i \rangle$ is the mean value of $G_i$, $\Delta$ represents the strength of fluctuations of the softness, and $\Gamma _{max}$ and $\Gamma _{min}$ are maximum and minimum of $\Gamma$ in the entire system. We set the mean softness parameter $\langle G_i \rangle = 1$. Since $\Gamma _{max} \approx -\Gamma _{min}$, a range of values for $G_i$ becomes $1-\Delta /2 \le G_i \le 1+\Delta /2$. Consequently, $\xi$ represents the correlation length of the fluctuations of $G_i$ and $\Delta$ represents the amplitude of these fluctuations. Additionally, $\xi$ and $\Delta$ are treated as variable independent parameters in this simulation, although the critical point argument for hyperuniformity suggests a potential interdependence between them^28^.

Taking into account the micro-deformation within the system, the interaction between the disks can be approximated using a harmonic potential. Disks *i* and *j* interact via a pairwise harmonic potential:

3 $$\begin{aligned} U(r_{ij})= & {} \frac{G_{ij}}{2} (r_{ij}-D)^2 f(D-r_{ij}) \end{aligned}$$

where $G_{ij} = (G_i + G_j) / 2$ and $r_{ij}$ is a center-to-center distance between disks *i* and *j*. Then we obtain

4 $$\begin{aligned} {\varvec{F}}_i= & {} \sum _j \frac{\partial U_{ij}}{\partial r_{ij}} \hat{{\varvec{r}}}_{ij} = -\sum _j G_{ij}(D-r_{ij}) f(D-r_{ij}) \hat{{\varvec{r}}}_{ij}, \end{aligned}$$

where $\sum _j$ signifies the summation over disk *j*, which is in contact with disk *i*. ${\varvec{F}}_i$ represents the force vector acting on disk *i*, while $\hat{{\varvec{r}}}_{ij}$ denotes the unit vector pointing from disk *i* to disk *j*. We specify that the interaction is purely repulsive, thus *f*(*r*) = $\Theta (r)$ where $\Theta$ is the Heaviside function. Additionally, we assume a loose packing in granular systems or soft components like emulsions and bubbles. Consequently, compression of the blob and overlap between pairs of disks are permissible. To streamline the model, friction between the disks is disregarded.

We compressed the system by 0.1% in both the *x* and *y* directions. Each particle follows the normalized overdamped equation.

5 $$\begin{aligned} d {\varvec{r}}_i = {\varvec{F}}_i dt, \end{aligned}$$

where $d {\varvec{r}}_i$represents the displacement of disk *i* during each simulation time step *dt*. We set *dt* = 0.001. We consider the system to have reached a steady state when the maximum velocity of all particles becomes less than 1.0$\times$ ${10}^{-6}$. We conducted 5 runs with the same $\xi$ and $\Delta$, but with the different planar distribution of *G*. We confirmed that the results remained essentially unchanged regardless of the initial conditions.

## Result

### Formation of the force network

**Figure 2:**
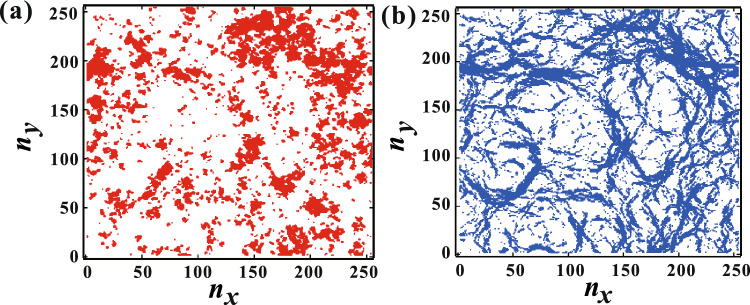
(**a**) planar distribution of the top 10% of $G_{ij}$ with $\xi = 10$ and $\Delta = 1$. (**b**) planar distribution of the top 10% of $F_{ij}$. In broad sections of the pattern, there appears to be a significant correlation between $F_{ij}$ and $G_{ij}$. Meanwhile, the distribution of $G_{ij}$ exhibits a cluster-like pattern, whereas the distribution of $F_{ij}$ forms a network.

**Figure 3:**
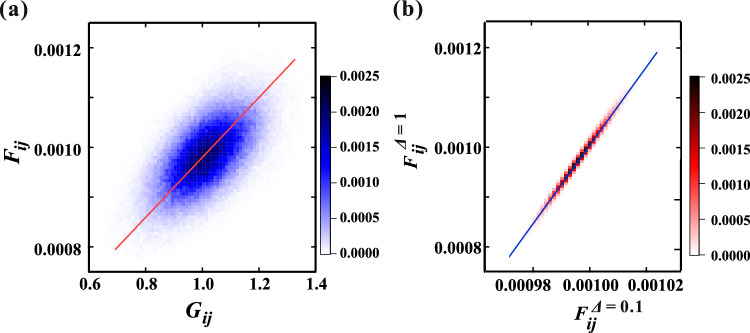
(**a**) Image plot of the probability of $F_{ij}$ and $G_{ij}$ at $\xi = 10$ and $\Delta = 1$. Upon fitting with a linear function, the obtained slope is 5.8 $\times$ 10^-4^, which is smaller than the slope when the displacements are uniform (1.0 $\times$ 10^-3^). It is observed that the data exhibit wide dispersion. (**b**) Image plot of the probability of $F_{ij}$ in a system with $\Delta$ = 0.1 and $F_{ij}$ with $\Delta$ = 1 for the same combination of *i* and *j*. The planar distribution of $G_{ij}$ remains unchanged. It is noted that the scattering of data is significantly narrower. The colors correspond to the probability.

Initially, we examined the force distribution. Figure 2a and b illustrate the planar distribution of the top 10% of $G_{ij}$ and the top 10% of the force $F_{ij}$ between disks *i* and *j*, respectively. The structure is generated using Eq. (1) with $\xi$ = 10 and $\Delta$ = 1 at *t* = 1000. For large sections of the pattern, there appears to be a significant correlation between $F_{ij}$ and $G_{ij}$. However, the distribution of $G_{ij}$ exhibits a cluster-like behavior, while that of $F_{ij}$ forms a thin network, which is a typical characteristic of amorphous materials^6–14^. Figure 3 presents an image plot of the probability of $F_{ij}$ and $G_{ij}$ at $\xi$ = 10 and $\Delta$ = 1. It indicates that $F_{ij}$ is qualitatively correlated with $G_{ij}$, although $F_{ij}$ is widely dispersed around a straight line. Fitting by a linear function yields a slope of 5.8 $\times$ 10^-4^. If the displacements of the disks were uniform, $F_{ij}$ and $G_{ij}$ would be perfectly proportional, and the slope would be 1.0 $\times$ 10^-3^. Hence, the smaller slope suggests spatially inhomogeneous displacement. Additionally, we compute the Pearson correlation coefficient *P* between $F_{ij}$ and $G_{ij}$. The obtained value of *P* = 0.61 indicates a correlation between $F_{ij}$ and $G_{ij}$, albeit not a strong one. This finding is consistent with the difference in planar distribution between $F_{ij}$ and $G_{ij}$. Further insight into the deviation of the correlation between $F_{ij}$ and $G_{ij}$ will be provided in the next subsection.

We also examined the force distribution when the planar distribution of $G_{ij}$ remains the same, but only the amplitude of the fluctuation $\Delta$ is varied. It was observed that the appearance of the planar distribution of $F_{ij}$ changes minimally. Figure 3b shows the correlation of $F_{ij}$ with the same combination of *i* and *j* at $\Delta$ = 0.1 and $\Delta$ = 1. The data scatter is significantly narrower, and it was found that the slope is 10.0, identical to the ratio of $\Delta$. Additionally, the Pearson correlation coefficient *P* = 0.994 for $F_{ij}$ between $\Delta$ = 0.1 and $\Delta$ = 1 is close to 1. This suggests that the planar distribution of $F_{ij}$ is primarily determined by the planar distribution of $G_{ij}$, rather than $\Delta$. Thus, the characteristic length of the force network is solely determined by $\xi$, which represents the characteristic length of *G* fluctuations.

### Sandwiched system

**Figure 4:**
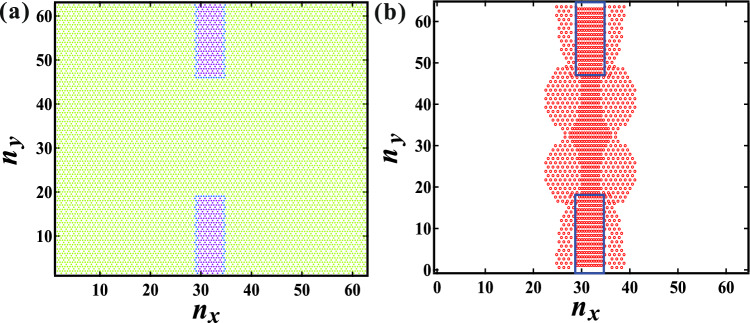
(**a**) The planar distribution of $G_i$, with a portion of the low $G_i$ (green) region being sandwiched by the high $G_i$ (purple) region. (**b**) The planar distribution of the top 10% of $F_{ij}$. The rectangular area enclosed by the solid line represents the high $G_i$ region. The force is prominently distributed not only within the high $G_i$ region but also within the low $G_i$ region that is sandwiched by the high $G_i$ region.

To explore the mechanism underlying the reduction in correlation between $F_{ij}$ and $G_{ij}$, numerical simulations were conducted using a model system. As depicted in Fig. 2, regions sandwiched by high $G_{ij}$ exhibit the larger $F_{ij}$. To simplify the analysis, a binary system was considered, comprising regions with high ($G_i = 1.5$) and low ($G_i = 0.5$) $G_i$ values. Figure 4a illustrates the planar distribution of $G_i$, with high $G_i$ regions positioned at the upper and lower centers, referred to as a “sandwiched system”. In this configuration, $n_x$ and $n_y$ are set to 64, with the width and height of each high $G_i$ region being 8 and 16, respectively. The system is subjected to a compression of 0.1%.

**Figure 5:**
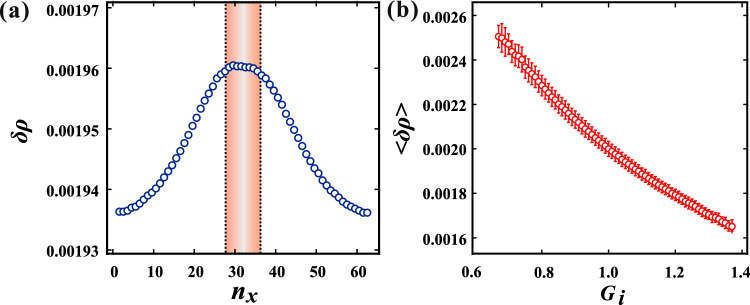
(**a**) The density change $\delta \rho$ profile as a function of *x* at the center along the *y*-axis ($n_y$ = 32) within the sandwiched system. Despite uniform $G_i$ values, notably higher density is observed in the region sandwiched by the high $G_i$ regions (within the dotted lines). (**b**) $\langle \delta \rho \rangle$ as a function of $G_i$ within the fluctuated system, as shown in Fig. 2. Error bars denote standard deviations. It is notable that $\langle \delta \rho \rangle$ is larger in regions with the lower $G_i$, while conversely, it is smaller in regions with the higher $G_i$.

Figure 4b illustrates the spatial distribution of the top 10% of $F_{ij}$ within the sandwiching system. The rectangular areas enclosed by solid lines denote the high $G_i$ regions. The force is distributed prominently not only within the high $G_i$ regions but also within the low $G_i$ region sandwiched by the high $G_i$ region. Previous studies have highlighted the crucial role of density change $\delta \rho$ in force distribution, given the strong dependence of bulk modulus on density in a jammed state^29^. Thus, we calculated the local density around disk *i*, denoted as $\rho _i$, utilizing the equation:

6 $$\begin{aligned} \rho _i \sim (D/\bar{r_{i}})^2, \end{aligned}$$

where $\bar{r_{i}}$ represents the mean value of $r_{ij}$ averaged over the nearest neighbor disk *j*. Subsequently, the density change is defined as $\delta \rho _i = \rho _i - \rho _0$, with $\rho _0$ (= 1) denoting the initial density. Figure 5a depicts the density change profile as a function of *x* at the center along the *y*-axis ($n_y$ = 32). The region sandwiched by the high $G_i$ regions is enclosed by dotted lines, as shown in Fig. 5. It is observed that the density within the sandwiched region surpasses that of other low $G_i$ regions. Consequently, elasticity within the sandwiched region effectively increases after compression, leading to significant force distribution.

Here we extend the findings derived from the model systems to the fluctuated system, as depicted in Fig. 2. Figure 5b shows $\langle \delta \rho \rangle$ plotted against $G_i$, where $\langle \delta \rho \rangle$ represents the mean value within the range $G_i - 0.005 < G_i \le G_i + 0.005$, with error bars indicating the standard deviation. It is observed that the density increases in the lower $G_i$ region, consistent with the density change observed in the sandwiched model. This observation suggests that the force network is constructed by connecting the higher $G_i$ regions.

### Total potential energy

**Figure 6:**
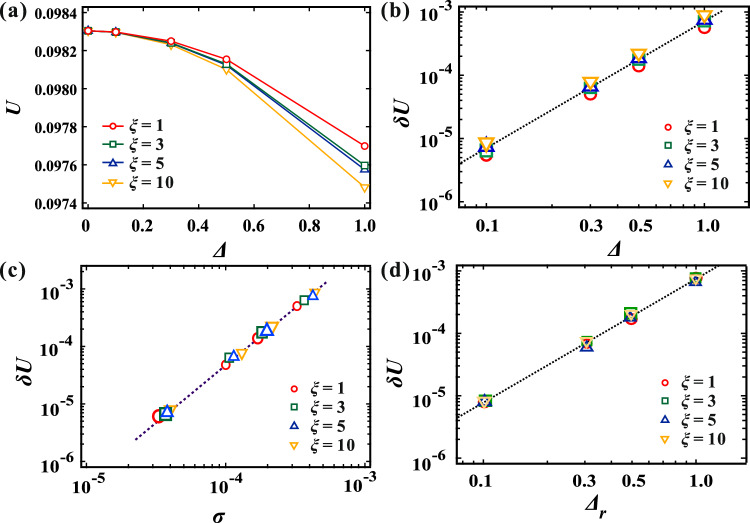
(**a**) The total potential energy *U* as a function of $\Delta$, showcasing various $\xi$ values. Circle, square, triangle, and inverted triangle symbols represent *U* with $\xi$ = 1, 3, 5, and 10, respectively. (**b**) The dependence of potential change $\delta U$ on $\Delta$. Notably, $\delta U$ exhibits a proportional relationship with $\Delta ^2$ for each $\xi$. (**c**) The potential change $\delta U$ as a function of $\sigma$, where $\sigma$ denotes the standard deviation of $\rho$. It is noteworthy that $\delta U$ across all systems with varying $\Delta$ and $\xi$ values adheres to a scaling law $\delta U \sim \sigma ^2$. (**d**) $\delta U$ is plotted against $\Delta _r$, illustrating collapsed behavior across different $\xi$ values when $\Delta _r$ remains consistent, despite variations in the $\xi$ and $\Delta$ combinations.

Although the underlying microscopic mechanism behind force chains has been unveiled, the impact of the force network on macroscopic physical properties remains ambiguous. In this study, we quantify the total potential energy within the systems, where the second derivative of the potential energy with respect to the volume corresponds to the bulk modulus. Figure 6a shows the variation of the total potential energy *U* with respect to $\Delta$. Different symbols such as circles, squares, triangles, and inverted triangles represent *U* values corresponding to $\xi$ = 1, 3, 5, and 10, respectively. It is observed that *U* predominantly relies on $\Delta$, with a minor dependency on $\xi$. This implies that the material undergoes softening when $\Delta$ is substantial. The influence of $\xi$ on *U* will be addressed subsequently.

To explore the relationship between the variation in *U* and the structural changes, we computed the potential change $\delta U = U_0 - U$, where $U_0$ represents the potential energy in a homogeneous system ($\Delta$ = 0). Figure 6b displays $\delta U$ plotted against $\Delta$. Our analysis reveals that $\delta U$ exhibits a proportional relationship with $\Delta ^2$ for each $\xi$ value. Given our previous demonstration of how the planar distribution of $G_i$ induces density heterogeneity, we further investigated the correlation between $\delta U$ and the standard deviation of density $\sigma$, as depicted in Fig. 6c. Notably, we observed that $\delta U$ across all systems with varying $\Delta$ and $\xi$ values conforms to a scaling law $\delta U \sim \sigma ^2$. This finding suggests that density change plays a crucial role in determining the total potential energy.

It is essential to recognize that the size of the blob is arbitrary and subject to ambiguity. The influence of this ambiguity on the coarse-grained particle model needs to be considered. Our investigation revealed that the size of the force network is determined by $\xi$, representing the characteristic length of planar softness inhomogeneity relative to the blob size. As such, changes in the definition of the blob size should correspondingly affect $\xi$. For instance, in a system with $\xi$ = 10, increasing the blob size by a factor of 10 should theoretically yield equivalence to a system with $\xi$ = 1. However, despite the scale change, slight discrepancies in $\delta U$ between $\xi$ = 10 and $\xi$ = 1 are observed, as shown in Fig. 6b. To address this discrepancy empirically, we introduce a rescaling factor $\Delta _r = (0.015 \xi +0.985) \Delta$, where $\Delta _r$ represents the rescaled $\Delta$. Specifically, we select $\xi$ and $\Delta$ values such that $\Delta _r$ matches for different combinations, such as $(\xi , \Delta ) = (1, 1)$ and $(\xi , \Delta ) = (10, 0.881)$. We find that $\delta U$ collapses across different $\xi$ values when $\Delta _r$ remains consistent, despite variations in the $\xi$ and $\Delta$ combinations, as shown in Fig. 6d. Thus, although the blob size remains ambiguous, achieving system equivalence through $\Delta _r$ rescaling demonstrates the robustness of this coarse-grained particle model.

### Theoretical approach

In this subsection, we present a theoretical elucidation of the mechanism behind the decrease in total potential energy with $\Delta$. Initially, we examine a simplified scenario involving a one-dimensional arrangement of three particles, labeled as disks *i*, *j*, and *k*, arranged sequentially. The interactions between disk *i* and *j*, and between disk *j* and *k*, are denoted by $U_{ij}$ and $U_{jk}$, respectively. The size of the disks is normalized to 1. We assume that the disk *i* is harder than the disk *k*. Under compression by $\alpha$, the distance between disk *i* and *j* contracts to $\alpha /2 - \delta$, while the distance between disk *j* and *k* contracts to $\alpha /2 + \delta$. Subsequently, the total potential energy can be expressed as $U = U_{ij}(\alpha /2 - \delta ) + U_{jk}(\alpha /2 + \delta )$. Expanding *U* to the second-order terms with respect to $\delta$, we obtain

7 $$\begin{aligned} U = U_{ij}(\alpha /2) + U_{jk}(\alpha /2) + [- U^{'}_{ij}(\alpha /2) + U^{'}_{jk}(\alpha /2) ]\delta + \frac{1}{2}[U^{''}_{ij}(\alpha /2) + U^{''}_{jk}(\alpha /2) ]\delta ^2, \end{aligned}$$

where $U^{'}$ and $U^{''}$ represent the first and second derivatives of *U* with respect to the distance, respectively. Meanwhile, by considering the balance of forces, we derived

8 $$\begin{aligned} U^{'}_{ij}(\alpha /2 - \delta )= & {} U^{'}_{jk}(\alpha /2 + \delta ). \end{aligned}$$

Expanding Eq. (8) to the first term with respect to $\delta$, we obtain

9 $$\begin{aligned} U^{'}_{ij}(\alpha /2) - U^{'}_{jk}(\alpha /2)= & {} [U^{''}_{ij}(\alpha /2) +U^{''}_{jk}(\alpha /2)] \delta . \end{aligned}$$

Subsequently, Eq. (9) is substituted into Eq. (7), resulting in:

10 $$\begin{aligned} U = U_{ij}(\alpha /2) + U_{jk}(\alpha /2) - \frac{1}{2}[U^{''}_{ij}(\alpha /2) + U^{''}_{jk}(\alpha /2) ]\delta ^2. \end{aligned}$$

Given that the second derivative of *U* is positive from the stability of the system, the total potential energy decreases $\delta \ne 0$. Finally, we substitute our potential, where $U_{ij} = \frac{1}{2} (\langle G \rangle + \Delta /2) (\alpha /2 - \delta )^2$ and $U_{jk} = \frac{1}{2}(\langle G \rangle - \Delta /2) (\alpha /2 + \delta )^2$, into Eq. (8).

11 $$\begin{aligned} \delta = \frac{\alpha }{4 \langle G \rangle } \Delta , \end{aligned}$$

then,

12 $$\begin{aligned} \delta U = \frac{\alpha ^2}{16\langle G \rangle }\Delta ^2. \end{aligned}$$

Thus, we obtained $\delta U \propto \Delta ^2$ in the simplified model.

Next, we explore the scaling law $\delta U \sim \Delta _r^2 \sim \sigma ^2$ in a two-dimensional system. The total potential energy can be expressed as the summation of Eq. (3) as follows

13 $$\begin{aligned} U = \frac{1}{2}\sum _i \sum _j \frac{1}{2}G_{ij}(D-r_{ij})^2. \end{aligned}$$

In a homogeneous system with a compression rate of $\alpha = 0.001$, the distance $r_{ij}$ between particles *i* and *j* uniformly becomes $D - \alpha D$, yielding a total potential energy of $U_0 = \langle G_{ij} \rangle (\alpha D)^2 N (Z/2) /2 = 0.098304$. Here, $Z (= 6)$ denotes the number of particles in contact with each particle. Subsequently, we consider the heterogeneous case where $G_{ij}$ and $r_{ij}$ are described as $G_{ij} = \langle G_{ij} \rangle + \delta G_{ij}$ and $r_{ij} = D - \alpha D + \delta r_{ij}$, where $\delta G_{ij}$ and $\delta r_{ij}$ are deviations from the average. Consequently, $\sum _i \sum _j \delta G_{ij} = 0$ and $\sum _i \sum _j \delta r_{ij} = 0$. Given that $\delta r_{ij} \sim 10^{-4}$, much smaller than $\alpha D$ and $\delta G_{ij}$, the term $\delta r_{ij}^2$ becomes negligible. Thus, $\delta U$ can be approximated as follows :

14 $$\begin{aligned} \delta U= & {} U_0 - \frac{1}{4}\sum _i\sum _j (\langle G_{ij} \rangle +\delta G_{ij})(\alpha D-\delta r_{ij})^2 \nonumber \\= & {} \frac{1}{2}\sum _i\sum _j \alpha D \delta G_{ij} \delta r_{ij} . \end{aligned}$$

In this context, when the correlation length $\xi$ exceeds 1, the value of $G_j$ for disk *j*, which is in contact with disk *i*, closely resembles $G_i$. Consequently, we posit that $\delta r_{ij}$ is approximately equal to $\overline{\delta r_i}$ and $\delta G_{ij}$ is approximately equal to $\overline{\delta G_i}$, where $\overline{\delta r_i}$ and $\overline{\delta G_i}$ denote the mean distance and the mean $G_{ij}$ averaged across neighboring disk *j*. Given the observation that higher values of $G_{ij}$ correspond to lower density or larger $\delta r_{ij}$, and conversely, smaller $\delta r_{ij}$ corresponds to lower $G_{ij}$, we establish the following relationship :

15 $$\begin{aligned} \overline{\delta r_i} \propto \overline{\delta G_i}. \end{aligned}$$

Here, $\Delta _r$ represents the difference of $G_i$, thus

16 $$\begin{aligned} \overline{\delta G_i} \propto \Delta _r. \end{aligned}$$

Finally, we derive

17 $$\begin{aligned} \delta U\approx & {} \frac{1}{2}\sum _i \alpha D \overline{\delta G_i} \ \overline{\delta r_i} \end{aligned}$$

18 $$\begin{aligned}\propto & {} \Delta _r^2. \end{aligned}$$

Expressed as $\sigma = \sqrt{\frac{1}{N} \sum _i (\rho _i^2 - \langle \rho \rangle )^2}$, the deviation in density can be formulated. Given the smallness of $\delta r_{ij}$ and $\alpha$, the expression can be approximated as follows

19 $$\begin{aligned} \sigma\sim & {} \sqrt{\frac{1}{N} \sum _i \left[ \frac{1}{Z} \sum _j \frac{2\delta r_{ij}}{D} \right] ^2} \nonumber \\\propto & {} \sqrt{\frac{1}{D^2}\sum _i \overline{\delta r_i}^2}. \end{aligned}$$

Combining Eqs. (15), (17), and (19), it is obtained that $\delta U \propto \sigma ^2$.

## Discussion

In our study, we compare our findings with prior vibration experiments, wherein the force network achieves homogeneity through vibration^9^. It is established in literature that the packing fraction of granular systems increases under vibration, approaching random close packing^1^. Notably, the state of random close packing is identified as hyperuniform, characterized by nearly uniform compressibility across all locations^28,30–32^. Our simulations corroborate that when $\xi$ is small, the propagation of force attains uniformity, aligning well with experimental observations.

Furthermore, previous studies have reported that the system exhibits a significant response to shear deformation near the jamming point, despite the lack of direct coupling between shear deformation and density changes, unlike compression. For instance, shear-induced decreases in the effective moduli of aggregates have been reported^33^. Additionally, fractures often occur following enhancements in density fluctuations induced by shear stress^22,23^. Investigating the behavior of systems characterized by inhomogeneous *G* under shear deformation would be of interest.

Additionally, we highlight the significance of the formation of the force network in controlling stress-induced phenomena. Research has shown that stress can suppress liquid-liquid phase separation in cells^34^ , and it can also influence the absorption and desorption of guest molecules in metal-organic frameworks (MOFs)^35,36^. Therefore, the characteristics of the force networks are expected to have a substantial impact on the functionality of these stress-induced phenomena. Consequently, material design based on this coarse-grained particle model is considered necessary.

## Conclusion

Many amorphous elastic materials, including glasses, emulsions, foams, and cells, exhibit complex macroscopic behaviors and are extensively utilized in everyday applications, necessitating both mechanical and chemical stability. Despite the recognized importance of the force network, the mechanisms governing its formation and its relationship with macroscopic properties remain unclear. Consequently, a qualitative understanding of the macroscopic properties of amorphous materials is still lacking. In such large systems, a phenomenological approach, such as a simple coarse-grained model, is generally effective. In this study, we investigate the formation of the force network and the variation in total potential energy using a coarse-grained particle model incorporating local packing fraction, particle shape, and microstructure into the softness *G* of the disk. We observe the formation of a force network resembling that observed in amorphous elastic materials, driven by the correlation length of fluctuations of *G*. Additionally, we find that amorphous elastic materials undergo softening due to the amplitude of the fluctuations of *G*, with softening scaling by the square of the density deviation, explicable through a simple theory. Furthermore, we observe that the ambiguity in blob size suggests the robustness of the coarse-grained particle model.

In this study, simulations using a coarse-grained particle model suggest that alterations in the effective local elastic modulus, prompted by changes in density, play a significant role. To validate local density changes and changes in local modulus of elasticity, it is essential to incorporate feedback from molecular dynamics simulations of compression. Additionally, we operated under the assumption that deformation is small in this simulation, thus applying the harmonic potential. However, it’s crucial to acknowledge that the potential may not remain harmonic under significant deformation. Although the decrease in total potential energy remains consistent, the relationship between total potential energy and density change could potentially vary. Therefore, exploring the potential dependence of our findings is a critical avenue for future research.

## Data Availability

All data generated or analyzed during this study are included in this published article.
